# Perspective on Wheat Yield and Quality with Reduced Nitrogen Supply

**DOI:** 10.1016/j.tplants.2018.08.012

**Published:** 2018-11

**Authors:** Christian Zörb, Uwe Ludewig, Malcolm J. Hawkesford

**Affiliations:** 1 Institute of Crop Science, Quality of Plant Products (340e), University of Hohenheim, 70593 Stuttgart, Schloss Westflügel, Germany; 2 Institute of Crop Science, Nutritional Crop Physiology (340h), University of Hohenheim, 70593 Stuttgart, Germany; 3 Rothamsted Research, Plant Sciences Department, Harpenden AL5 2JQ, UK

**Keywords:** wheat, nitrogen, quality, yield, environment, nitrogen use efficiency, NUE

## Abstract

Wheat is an important cereal crop with a high demand for nitrogen (N) fertilizer to enable the grain protein accumulation that is necessary for baking and processing quality. Here, perspectives for the development of improved wheat genotypes with higher yield stability, better grain quality, and improved N use efficiency to lower environmental impacts are discussed. The development of improved wheat genotypes, for example, genotypes that lack storage proteins that do not contribute to baking quality (e.g., by genome editing), in combination with appropriate N fertilizer management to prevent N losses into the environment underpins a novel approach to improving N use efficiency. This approach may be particularly applicable to wheats grown for animal feed, which have lower quality and functionality requirements.

## Highlights

Wheat production has been criticized because of a Janus-faced nature for the use of nitrogen (N) fertilizer: it beneficially produces high quality and/or high yields but also has potential negative environmental impacts.

Crop production and N management therefore present a sustainability dilemma. A high N application is obligatory because high wheat protein content is important for baking quality, but simultaneously, high N may cause environmental N pollution via leaching or gaseous N losses.

Solutions involve both the optimization of the management of N fertilizer and breeding strategies to improve N use efficiency of the crops.

A conventional but relatively easily achievable solution would be improving yield stability as a critical factor to improve N use efficiency. A novel approach may be to manipulate wheat grain N demand by selectively reducing some storage proteins to help reduce N fertilizer inputs.

## Wheat Production and Use: Recent Problems

Wheat is the second most widely grown crop in the world, estimated at 200 million ha. Wheat grain consumption accounts for 19% of the calories in the global human diet [1], while about 40% of wheat produced is fed to poultry and livestock. Wheat grain is rich in carbohydrates and has a higher protein content than other major cereals, such as rice (*Oryza sativa*), maize (*Zea mays*), rye (*Secale cereale*), and millet (*Pennisetum glaucum*) [2]. It also contains substantial amounts of minerals (e.g., Zn, Fe), vitamins, and phytochemicals, making it a good source of nutrition 3, 4, 5, 6. Wheat is used globally for the production of bread, pasta, and other bakery products and to a small extent for industrial products.

There is an absolute requirement for N for wheat growth, and crop yield and quality depend upon substantial N inputs. Initially, this drives canopy formation required for photosynthesis that, in turn, drives yield. Subsequently, the major sink is the reproductive component, namely, the wheat grain. More than half of industrially fixed N is used by agriculture, worldwide amounting to in excess of 180 Mt/year [7]. The production and transport of N fertilizers by the Haber–Bosch process is highly energy intensive and depends on fossil fuels; however, the costs for the fertilizer for many farmers continue to be relatively low, in some cases due to state subsidies. Ready availability and cheap supply encourage overuse, and environmental problems in some agro-ecosystems remain an issue.

N losses from the production system occur as nitrate (NO_3_^−^) leaching or as the gaseous products of denitrification in soil: futile di-nitrogen (N_2_); nitrous oxide, a greenhouse gas with 300 times the heat-trapping capacity of carbon dioxide (CO_2_); and finally ammonia. Except for N_2_, these gases contribute to pollution and climate change [8]. Furthermore, N accumulates in the soil. N losses reduce ecosystem productivity and biological diversity and contribute to eutrophication. As a consequence, maximal acceptable NO_3_^−^ level in the European Union (EU) in freshwater resources is legislated to 50 μg/l. This target, however, is not achieved in some EU areas, especially those with high organic manure inputs; the disposal of N-containing manure from locally concentrated animal farming industry may be a further future threat for these regions. The environmental problems associated with N inputs continue to be an issue in many parts and countries of the world, including the USA and large regions of China. Although, substantial progress has recently been made, wheat remains the least N use efficient (NUE) major crop, whereas NUEs of maize and rice are around 25% higher [9]. Fertilizer regulations to improve N use management with the clear goal to decrease N inputs and losses into the environment differ in each country. However, major concerns from farmers are that yield and grain quality will not be maintained at high levels if N fertilizer use is substantially reduced. This review article provides background information and some figures for what is achievable in future wheat production as well as a concept for an improved strategy, by using new wheat genotypes with higher NUE to maintain or even improve grain yield, yield stability, and quality.

## Modern Wheat Fertilizer Management

Globally, a growing population and per capita increasing income will translate into greater food and protein needs, using a nearly static land area that relies on intensive management of agricultural inputs. Over the past 30 years, there has been a positive correlation between cereal production and N fertilizer use (both from mineral fertilizer and organic recycling fertilizer) in developing countries. Synthetic N fertilizer use worldwide was at only 9.2 Mt N in 1960; it increased to 80.4 Mt N in 1995 and since then has steadily increased to 108 Mt N in 2015 [10]. Global analysis identified that in the past five decades the N use efficiency in many countries first decreased and then increased with economic growth. Despite many county-specific socio-economies and N pollution avoidance policies, the total N use efficiency, and especially that of wheat systems, remains relatively low [11]. Overall, crop NUE on a worldwide scale is 47% [12] [here defined as (total grain N removed – N coming from soil]/fertilizer N applied)] and for some decades was substantially lower for cereals (33%) 12, 13.

**Figure 1:**
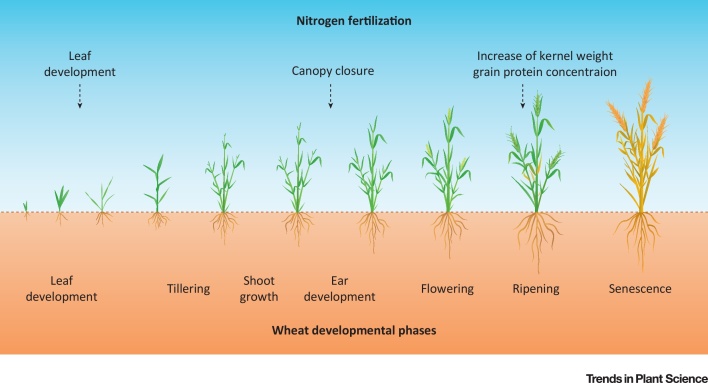
Wheat Developmental Phases. Nitrogen (N) fertilizer application management of wheat during the vegetation period and influences on grain yield and quality parameters. Fertilization, marked with arrows.

Wheat has three main phases of growth with considerable N demands (Figure 1). After sowing, the approximate 6 mg of total protein reserves of the kernel is sufficient to maintain the germination and growth of the seedling until the first leaf emerges. Further N has to be acquired by the root system, but at this stage the root is very small. Thus, additional fertilizer might be best applied directly as a small leaching-resistant ammonium placement below the seed row, but only if there is insufficient N from mineralization available. Despite global wheat NUE being apparently poor, root N uptake of individual wheat plants, mostly in the form of NO_3_^−^, ammonium, or even urea [14], is generally very efficient. Until now, there is not a single example that agronomic NUE of the best-performing crop varieties (i.e., those that are grown by farmers) was further improved by genetic targeting of uptake systems or primary N assimilation. Breeding progress over several decades in the UK, Argentina, and Italy did not increase the N uptake per unit root length and reduced root length density [15]. However, five decades of selection for yield reduced root length density and increased N uptake per unit root length in Australian wheat varieties. For wheat to obtain high yield and quality in a humid Northern Hemisphere, a first rate of N fertilizer application (up to 60 kg N ha^−1^) is considered necessary at the end of winter, around growth stage 31 (ear at 1 cm; [16]), before the second leaf emerges (Figure 1). A second application (up to 60 kg N ha^−1^) is at tillering. Within that, the N concentration in the tissue is responsible for the formation of the numbers of tillers per plant. N-accumulating genotypes that later translocate N (as a reserve for later grain filling) to the grain appear highly efficient in low N conditions, but the NUE in well fertilized conditions remains poor [17]. The third application of N fertilizer application is before growth stage 37 (flag leaf just visible), and the aim is to promote protein buildup in the ears 18, 19. Currently, in modern wheat varieties, the grain protein concentration is required to be above 12% dry matter, which means that amino acids must be synthesized in high amounts in vegetative tissues and transported to the developing grain, where storage proteins are formed (Figure 1). This process is only moderately influenced by late application of high amounts of N fertilizer (up to 150 kg N ha^−1^), as root activity declines during maturation and may thus be responsible for the environmental problem of N losses. Excessive use of fertilizer N with total average N application rates up to >500 kg N ha^−1^ for winter wheat is getting rare, even in China [20]. Such high N rates greatly will inevitably lead to large losses of N 21, 22. However, if N rates are massively reduced, the wheat plant cannot exploit the genetic potential to build up as much as possible protein during kernel development [23]. However, recent farm trials in southern Germany showed that single N applications of various fertilizer forms after tillering gave the same high yield and grain protein concentrations, without increased risk of NO_3_^−^ leaching, as split applications, probably because of large soil stocks due to substantial N depositions over decades [24]. The flag leaf is mainly important for N assimilation and serves as a main source for N metabolites such as amino acids that are subsequently transported into the developing kernels [25]. The N availability in the soil solution surrounding the root at this developmental phase is crucial for exploiting the genetic potential for protein buildup in the kernels. In organic management systems, the high availability of N at this specific period of growth (anthesis) is not easily manageable. Although it may not be sufficient to ensure the target protein concentration, soil organic N mineralization usually reaches a peak during the grain filling period because of higher temperatures compared with the vegetative growth phase (Figure 1). Therefore, the protein concentration in grains of organic farming systems, in general, is some percent lower (up to 40%) compared to that of conventional agriculture 3, 4. Thus, organic agriculture is a way to produce wheat grain with a lower N loss but that goes along with the handicap of 20–40% lower yield and the risk of lower grain protein concentration [3]. Therefore, protein quantity in grains may differ in both management systems. Moreover, protein quality varies due to the different management systems, even when the same wheat genotypes are used 3, 26. Therefore, prices for these products must be 20–40% higher compared to conventional wheat grain to be profitable; however, price is determined by what people are willing to pay.

## Modern Wheat Genotypes

Wheat is a polyploid, combining three genomes from grasses: an *Agilops* species that brought the BB genome, *Triticum uratu* that brought the AA genome, and *Triticum tauschii* that brought the DD genome and has been cultivated for 10 000 years. The AABBDD hexaploid genome mix of our modern wheat varieties includes bread wheat, as well as spelt 5, 27. Another widely used wheat form is *Triticum durum* with an AABB genome, which is useful for hard wheat products such as pasta and other products around the Mediterranean basin. Modern wheat varieties may be classified as winter wheat. Winter wheat is sown in autumn and is relatively frost resistant. Spring wheat, which is sown in spring, usually has lower yields and lower protein concentrations than winter wheat. Winter wheat can be used for bread making. There are some hundreds of modern wheat varieties that might be used in a certain country, be suitable in a special climate, or be suitable for a different soil type or for specific products. Apart from yield, high resistance against fungal diseases and high grain protein concentration are major targets of modern wheat breeding. While wheat hybrids (compared to lines) do not appear to have the large yield advantage from heterosis that is found in maize, yield stability and potentially quality traits benefit from crossing lines of different heterotic groups 28, 29. Varieties with higher yield stability may provide an overall benefit with respect to N losses, because farmers project and apply the seasonal fertilizer requirement before knowing whether weather conditions allow the genotype to fully retrieve its yield potential. Thus, stabile higher yielding varieties prevent excess losses in unfavorable years.

**Figure 2:**
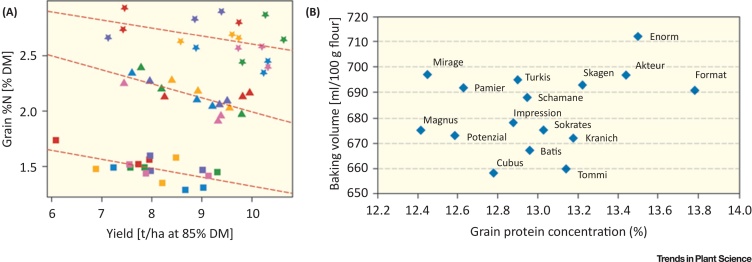
Relationship of Yield with Grain Protein Concentration. (A) Negative correlation of single-year grain nitrogen (N) concentrations of six British wheat varieties (different colors), with yield at three N levels: 100 kg/ha (squares), 200 kg/ha (triangles), and 250 kg/ha (open squares) [30]. DM, dry matter. (B) Scatterplot of grain protein concentration and baking volume of loaf after a standard baking test (rapid mix test) for 1 kg of flour (bread wheat). Northern Europe winter wheat varieties 33, 34.

In northern European countries such as Germany and the UK, wheat grains are rated according to protein concentration, and growers are paid according to the grain protein concentration. There are mills and bakeries that require a minimum of 12.8% or even higher protein concentration in wheat flour. This protein concentration requires high fertilizer rates after anthesis, for high buildup of storage protein. Despite large efforts by breeders, the negative relationship of yield with grain protein concentration is difficult to break (Figure 2A) [30], although grain quality of modern varieties has increased by enhancing storage protein concentration (measured by near infrared spectroscopy-N content) in the grain (Figure 2B). Because of increased N fertilizer and genotypic improvement, the protein concentration in grains, on average, in German wheat genotypes rose from about 7–8% crude protein in the 1960s to 12–16% in modern genotypes (Figure 2B) [31]. There is a limitation for increasing N in grain further because of the inverse relation of yield and protein concentration, which leads to high-yielding genotypes on the one hand and high-quality genotypes, as defined by high protein content, on the other hand (Figure 2). Although, some authors have suggested that grain protein concentration may be energy limited [32], there is no clear indication that this is true for wheat. Therefore, farmers have to decide which market to target: the bread-making market with high quality or the animal feed market with high yield. If a farmer decides to produce bread wheat, he or she needs to use varieties with high baking potential, which in the past decades equated with high raw protein content.

However, some new varieties with a high raw protein content (13–16%) performed equally (baking volume) compared to other varieties with a 1–2% lesser protein content (Figure 2B) 33, 34. It may be concluded that (i) the strategy of increasing raw protein content to achieve higher baking quality is exhausted and (ii) farmers produce high raw protein by high N application, which inevitably results in high environmental losses, without any increase in grain quality. This general practice is a waste of N. The solution is to change the premium payments systems in such countries (Germany, UK) towards a system that quantifies protein fractions that contribute to grain quality (discussed in the next section), rather than just raw protein concentration. The testing method has to be fast, inexpensive, and sensitive and may involve ‘lab-on-a-chip’ [35] or asymmetric flow field-flow fractionation methods [36] to quantify the amount of gluten macro polymers, but suitable rapid and cheap spectroscopic methods should also be developed.

## The Problem: Two Main Factors Affecting Protein Concentration and Composition

### Effect of Genetic Background on Protein Concentration and Composition

**Figure 3:**
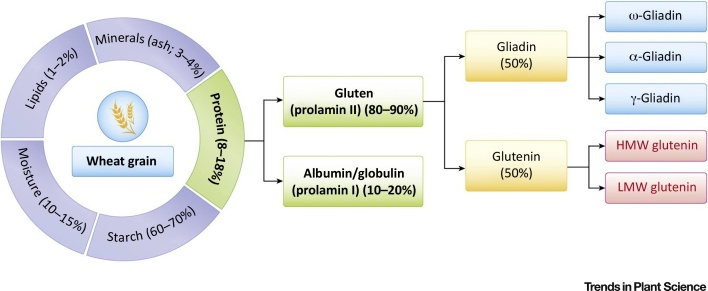
Main Components of Wheat Kernels and Main Storage Protein Fractions. Sulfur-rich proteins (α, γ, and LMW) that are contributing with S–H bridges to the rheology of the dough-making process. HMW, high molecular weight; LMW, low molecular weight.

Transcriptome studies have shown that more than 30 000 genes are expressed in the developing wheat grain [37], while proteomic analysis of mature grain has identified about 1125 individual components [38]. However, many of these components are present in small amounts and have little or no impact on the use of the grain. One protein fraction, the prolamin storage proteins, correspond to the gluten proteins and are dominant in terms of amount and impact [5] (Figure 3). The precise number of individual gluten protein components has not been determined; together, they have been estimated to account for about 80% of the total grain protein in European wheats [39]. Gliadin proteins contribute mainly to the viscosity of the dough, whereas glutenin proteins contribute with the elasticity of the dough (Figure 3). The existence or absence of some kinds of allelic variants of high molecular weight glutenin subunits (HMW-GS) is correlated with quality of baking bread [40]. HMW-GS 5 + 10 at the Glu-D1 locus is connected with a higher quality of baking bread than HMW-GS 2 + 12 [41]. An extra cysteine group on 1Dx5 compared to 1Dx2 results in a higher quality of wheat cultivars containing HMW subunit pair 5 + 10, which allows development of a supplement disulfide link and larger size of the polymer [42]. Moreover, cultivars and landraces that contain more of Glu1Bx7 (Bx7OE) have enhanced strength of dough 43, 44. Cultivars of bread wheat express between three and five HMW subunit genes, with the encoded proteins accounting for up to 12% of the total grain protein. Breeding progress in wheat has started to consider gluten fractions [45]. However, there is another storage protein fraction, the albumin/globulin fraction (Figure 3), that is considered unimportant for baking quality; the albumin/globulin proteins consist of structural grain N and sulfur storage proteins, mostly with unknown function [46]. This protein fraction accounts for up to 20% of the total N in grain [47] and theoretically may be reduced without loss of baking quality.

### N Fertilizer Application Effects in Wheat Production and Quality

N is the most important plant nutrient in terms of yield formation. Moreover, N plays the most important role in determining the concentration of storage protein in grains. Studies show control of grain N accumulation by the level of N fertilizers for wheat 19, 48, 49, with grain protein concentration increasing by increasing N fertilizer input 50, 51.

Environmental conditions such as fertilizer management have a notable impact on baking quality of wheat flour by influencing the concentration and composition of gluten protein subunits and their assembly and polymerization. Weather conditions and water availability are also important factors influencing storage proteins 52, 53. At future elevated CO_2_ levels, grain protein concentrations of current wheat varieties will inevitably decrease by 6–8% [62] A recent FACE experiment also showed that NO_3_^−^-based fertilization was superior to ammonium fertilizers, and even with elevated CO_2_, N uptake efficiency in the field was close to 100% at medium N supply but around 75% with luxury N supply [54].

As discussed above, grain N is obtained via two pathways: remobilized N from the canopy (leaves and stems) and up to 50% from soil after anthesis 55, 56 (Figure 1). Hence, to increase of grain protein, ideally both N sources need to be boosted at an appropriate rate with a suitable type of fertilizer. Moreover, the amount of N fertilizer application changes not only quantity but also quality (composition) of grain proteins [57]. It was suggested that the ω-gliadins and HMW-GS, which are rich in glutamine and proline and are the metabolically most inexpensive amino acids, may be good sinks for N, when there is N surplus [53]. Interestingly, increasing the rate of N fertilizers in 13 wheat varieties did not affect the amount and composition of albumins and globulins, but the concentration of ω-gliadins and HMW-GS increased [50]. In addition, the effect on gliadins was more pronounced than on glutenins, as well as the effect on major protein types (α- and γ-gliadins and LMW-GS) in comparison with minor types (ω-gliadins, HMW-GS). Other studies showed that type and rate of N fertilizers can change the ratio of HMW-GS to LMW-GS 50, 58, 59. Genetic downregulation of the gliadin fraction by RNAi was highly efficient, and different N fertilization had substantial effects on other gluten proteins 60, 61. These studies demonstrate the impact of N fertilizer management on the quantity and quality of wheat grain protein.

## Concluding Remarks and Future Perspectives

The future (see Outstanding Questions) of wheat production needs to be coordinated with a considerable reduction of environmental N losses. This requires a reduction in N fertilizer application, and several solutions are proposed:

(i) The reduction of use of N-based fertilizers (up to 20–40%) without quality and quantity loss might be achievable. The reduction has several beneficial effects, such as reducing energy for fertilizer production mostly by Haber–Bosch method, reducing costs for farmers, and reducing negative environmental effects of leached and gaseous N, which was not taken up by plants. Another aspect to reduce the amount N fertilizer is to adapt the fertilizer application management or to optimize the timing and rates of N fertilizers on top of current large soil N deposits resulting from decades of high N fertilizer rates and high mineralization potential, with a focus during development of the ears (see above) 51, 54.
(ii) Using wheat genetics for the deletion of excess storage protein genes that may not contribute to baking quality or are even harmful for consumers with wheat allergy or celiac disease, but consume N resources. Targets of these N-consuming proteins are many proteins from the albumin/globulin fraction, as well as to a lesser amount the prolamins. Such genes, which do not directly contribute to product quality, have to be identified and reduced. This can already be done by classical wheat breeding. By contrast, deleting genes without trace of transgenic gene sequences in the product is possible by genome editing techniques such as CRISPR-Cas. Although mutants produced by targeted genome editing that do not contain any residual transgene in the genome should be acceptable for the public, these have just recently been classified as being genetically modified by the European Court of Justice, so strong regulations apply. However, there is no global consensus, and there may be different views on these genome editing techniques in America and other parts of the world. A prerequisite of this strategy, to delete these genes, is that these storage genes are really dispensable. As mentioned above, certain genes (1Dx2, 1Dx5, etc.) and their functions are well known, but many others of the prolamin fraction, as well as those of the gliadin/glutenin fraction, still have to be characterized. One could argue that grains need these (general) storage proteins to maintain their germination vigor. However, it is already technically possible to delete a whole set of storage proteins such as α-gliadins while maintaining the vigor of wheat growth 58, 59. In this case, no effects on flour functionality and slightly detrimental effects on baking quality were noted; however, more substantial alterations would be expected to seriously modify baking properties. A successful commercial strategy using this approach would need to balance reductions in storage proteins with requirements for end use and, for example, may be best applied to feed wheats rather than bread-making wheats.
(iii) The most effective approach will be to use strategies simultaneously: the use of wheat varieties without unnecessary storage protein genes and use of an optimized fertilizer application strategy.
(iv) Finally, the focus on more yield-stable varieties will prevent N losses in unfavorable years for all strategies, with or without modifications to storage protein composition.

In summary, there is potential by using these agronomic and biotechnological strategies to avoid unnecessary N fertilizer use in the future, to decrease environmental impacts, save money and energy, and still produce quality wheat.

Outstanding Questions Is there a potential to decrease nitrogen fertilizer in wheat production worldwide?

Can we optimize nitrogen fertilizer application strategy?

Which wheat storage proteins contribute to wheat (baking) quality?

Which wheat storage proteins do not contribute to quality?

Can we improve plants by deleting such storage proteins that do not contribute to quality and thereby reduce nitrogen supply in wheat cultivation substantially?
